# Effectiveness of a behavioural intervention to prevent excessive weight gain during infancy (The *Baby Milk* Trial): study protocol for a randomised controlled trial

**DOI:** 10.1186/s13063-015-0941-5

**Published:** 2015-10-06

**Authors:** Rajalakshmi Lakshman, Fiona Whittle, Wendy Hardeman, Marc Suhrcke, Ed Wilson, Simon Griffin, Ken K Ong

**Affiliations:** MRC Epidemiology Unit and UKCRC Centre of Excellence in Diet and Activity Research (CEDAR), University of Cambridge, Cambridge, UK; Behavioural Science Group, Department of Public Health and Primary Care, University of Cambridge, Cambridge, UK; Centre for Health Economics, University of York, York, UK; Cambridge Centre for Health Services Research, Department of Public Health and Primary Care, University of Cambridge, Cambridge, UK

**Keywords:** Infancy, Formula-milk, Obesity, Weight gain, Prevention, Behavioural change, RCT

## Abstract

**Background:**

Infancy is a period of rapid growth and habit formation and hence could be a critical period for obesity prevention. Excess weight gain during infancy is associated with later obesity and formula-fed babies are more likely to gain excess weight compared to breastfed babies. The primary trial outcome is a change in the weight standard deviation score from birth to 1 year.

**Methods/Design:**

We will recruit 650 to 700 parents who introduce formula-milk feeds within 14 weeks of their baby's birth to a single (assessor) blind, parallel group, individually randomised controlled trial. The focus of the intervention is the caregiver (usually the mother), and the focus of the primary outcome is the infant. The intervention group will receive the behavioural intervention, which aims to reduce formula-milk intake, promote responsive feeding and healthy weaning, and prevent excessive weight gain during infancy. The intervention is based on Social Cognitive Theory and action planning (‘implementation intentions’). It consists of three components: (1) a motivational component to strengthen parents’ motivation to follow the *Baby Milk* feeding guidelines, (2) an action planning component to help translate motivation into action, and (3) a coping planning component to help parents deal with difficult situations. It will be delivered by trained facilitators (research nurses) over 6 months through three face-to-face contacts, two telephone contacts and written materials. The control group will have the same number of contacts with facilitators, and general issues about feeding will be discussed. Anthropometric outcomes will be measured by trained research staff, blind to group allocation, at baseline, 6 months and 12 months following standard operating procedures. Validated questionnaires will assess milk intake, temperament, appetite, sleep, maternal quality of life and maternal psychological factors. A 4-day food diary will be completed at 8 months.

**Discussion:**

The results of the trial will help to inform infant feeding guidelines and to understand the links between infant feeding, behaviour, appetite and growth.

**Trial registration:**

ISRTCN20814693. Registration date 13 January 2011.

## Background

Childhood obesity has important consequences for morbidity and mortality in childhood and in later life [1]. In 2010, 43 million children under the age of five were obese or overweight worldwide and the prevalence is predicted to rise from 6.7 % to 9.1 % in 2020 [2]. In England, data from the National Child Measurement Programme (NCMP) show that by the time children start school, more than one in five are overweight, and one in 10 are obese [3]. Hence, tackling childhood obesity through focussing on the early years has become a national priority [4]. However, to date, there is little evidence on which to develop effective strategies to prevent childhood obesity [5]. A Cochrane Review identified 22 randomised trials to prevent childhood obesity; although some diet and physical activity interventions were effective in promoting a healthy diet and increased physical activity levels, they were not on the whole effective in preventing weight gain [6]. Only one of the included studies recruited children below one year of age (average age 21 months, *n* = 43), and that was a home-visiting programme for high-risk Native-American children, focussing on improving parenting skills to develop appropriate eating and exercise behaviours to prevent obesity [7].

Infancy is a period of rapid growth and could be a critical time when obesity prevention may be most effective. Recent systematic reviews have described a consistent association between rapid weight gain in infancy and subsequent obesity risk in childhood and later life [8–10]. Energy deposition as a percentage of total energy requirements decreases from 40 % at 1 month to 1 to 2 % from 12 months until mid-adolescence. Therefore, infancy weight gain is more closely related to energy intake, than is weight gain in childhood or in later life. In 2004, based on new data on energy expenditure in infants, the World Health Organisation (WHO) reduced the recommended energy requirements for infants by 15 to 20 % [11]. Current formula-feeding instructions in UK are based on the previous 1985 WHO recommendations and this may be an important reason why formula-fed babies are at a greater risk of rapid weight gain and subsequent obesity than breastfed infants. Three meta-analyses of observational studies reported that obesity risk at school age was reduced by 15–20 % in breastfed compared with formula-fed infants [12–14]. In 2006, the WHO published growth charts based on the growth of breastfed babies and recommends this lower plane of growth as the optimum standard for growth of all infants [15, 16]. While the benefits of breastfeeding are well recognised, the 2010 UK Infant Feeding Survey showed that only 21 % of babies were exclusively breastfed at age 6 weeks and 1 % at 6 months [17]. Hence in addition to supporting breastfeeding, it is important to optimise the growth of formula-fed babies.

In the *Baby Milk* trial we aim to evaluate the efficacy, cost-effectiveness and acceptability of a theory-based, multi-component intervention to reduce formula-milk intake (based on the 2004 WHO recommendations for energy requirements) and prevent excess weight gain during infancy. We also aim to understand the psychological mediators of any effect of the intervention on formula-milk and energy intake.

## Methods/Design

### Study design

The *Baby Milk* trial is an explanatory, parallel, individually randomised controlled trial of parents (mainly mothers) and their babies who are formula-fed. The focus of the primary outcome measurement is the baby, but the focus of the intervention is the caregiver. The intervention will commence when the baby is enrolled in the study (between 2 and 14 weeks) and be delivered up to 6 months. Babies will be followed up to age 1 year. In this explanatory trial, the intervention programme will be delivered by trained and quality-assured facilitators (research nurses). Participants in the control group will be offered the same number of contacts and standard advice about formula feeding, and weaning will be discussed.

Ethical approval has been obtained from the Cambridgeshire 4 Research Ethics Committee (Ref:10/H0305/9), and fully informed written consent will be taken from all participants.

### Recruitment and retention

Parents and their babies who are partially or fully formula-feeding within 14 weeks of birth will be identified by research staff on a postnatal hospital ward; health professionals, including GPs, midwives and health visitors; and via a mail-out using the National Health Service (NHS) database (SystmOne). Most babies in England are delivered in the hospital and looked after by a community midwife for the first 2 weeks after discharge from hospital. Subsequently, they come under the care of a health visitor who records if they are formula-feeding in the NHS database. This database will be used to send a participant information sheet to parents in the catchment area. Research staff and midwives will identify babies within 2 weeks of birth. General Practices (GP, nurse, and other practice staff) will invite participants at the baby’s 6-week health check and immunisation clinics. Parents who return a reply slip expressing an interest in taking part or finding out more about the study will be contacted by research staff at the Medical Research Council (MRC) Epidemiology Unit to answer any questions, check eligibility criteria, and where appropriate, invite them for their baseline visit (at 2 to 14 weeks age). GPs and health visitors will be informed by letter if a baby under their care has been recruited into the study.

Maximising retention is an important issue and regular (4 to 6 weekly) contacts with participants in both groups should reduce attrition. The information sheet will emphasise the importance of follow-up irrespective of group allocation and adherence to the intervention. Participants who drop out from the intervention will be encouraged to attend for outcome measurements. For participants who withdraw from the trial, any data collected up to the withdrawal date will be retained and safely stored, but contact details will be removed.

### Inclusion and exclusion criteria

All healthy, term infants who are receiving formula-milk within 14 weeks of birth will be eligible to participate. We will exclude low birth weight (<2,500 g) and pre-term infants (<37 weeks gestation) and infants with major malformations, hormonal or metabolic diseases that might interfere with nutrition or growth. Infants who are on special formulas (soya-based, lactose-free, hydrolysed or anti-reflux formulas) at the time of recruitment will also be excluded; however, they will not be excluded if they are put on a special formula after randomisation.

### Measurements

A summary of the measurements undertaken is presented in Table 1.

**Table 1:** Study measures

Measures	Baseline	4 months	6 months	8 months	12 months
Questionnaire measures					
Baseline questionnaire^a^	I, C				
Milk feeds questionnaire	I, C	I, C	I, C		
Maternal attitudes^b^	I, C		I, C		
Temperament, sleep and eating behaviour^c^			I, C		
4-day diet diary^d^				I, C	
Health Service Utilisation			I,C		I, C
Maternal quality of life^e^	I, C		I,C		I,C
Maternal anxiety^f^	I, C		I,C		
Intervention evaluation			I		I
Anthropometry					
Parents’ height	I, C				
Parents’ weight	I, C		I, C		I, C
Baby weight	I, C	I	I, C		I, C
Baby supine length	I, C	I	I, C		I, C
Baby head circumference	I, C		I, C		I, C
Baby abdominal circumference	I, C		I, C		I, C
Baby skin fold thickness					I, C
Baby abdominal Ultrasound					I, C

*I* Intervention group; *C* Control group

^a^Baseline Questionnaire: Parents: Demography (age; education - age at completion and highest qualification; occupation; ethnicity; marital status - cohabiting/married/single); weight and height. Mother’s: lifestyle (smoking - during pregnancy, current alcohol consumption), pregnancy (duration, weight -before pregnancy and at time of delivery), delivery (normal, instrumental, or caesarean) and previous children (number, feeding-breast, formula, dependent on instructions/appetite/growth - Likert scale). Baby’s: sex, weight and length at birth and 6 weeks

^b^Maternal attitudes and feeding practices (questionnaire developed for use in this study): Mother’s attitudes; psychological measures based on Social Cognitive Theory (self-efficacy, outcome expectancy, intentions, and motivation); perception of baby’s weight. Type of feed (breast/ expressed/ formula/ mixed), feed on demand/routine/both; feed based on instructions/appetite/growth – Likert scale

Bottle feeds (type, frequency, amounts, duration, how reconstituted, advice followed, age when started), Breastfeeds (frequency, duration, and expressed milk). Other drinks and foods. Age at weaning (only at 6 months)

^c^Validated questionnaires used with permission from Prof Jane Wardle and Mary Rothbart

^d^Diet diary: developed in collaboration with MRC HNR, and used in the National Diet and Nutrition Survey of Infants and Young Children

^e^Quality of Life: EuroQol Visual Analogue Scale and Short Form 8

^f^Anxiety: Spiegelberger Short State Anxiety Inventory

#### Anthropometry

Anthropometry data will be collected by trained research assistants using standard operating procedures. In order to minimise bias, the measurement team will be blind to group allocation and will be trained to avoid discussing this with parents. Parents will also be advised not to discuss allocation with the measurement team.

Weight will be measured with the baby undressed, using Seca Infant Electronic Scales™ and recorded to the nearest 0.01 kg. For supine length, the baby will placed on a Kiddimeter™ or Starters mat™, with only the nappy on and measured to the nearest 0.5 cm. Abdominal waist circumference will be measured using a D-loop non-stretch fibreglass tape measure and head circumference will be measured using the Child Growth Foundation reusable tape. Sub-scapular, triceps, quadriceps and flank skin fold thickness will be measured using the Holtain Tanner™/Whitehouse™ skin fold calliper with an average of three measurements taken for each site. A standard ultrasound device with a 3C-RS curved transducer will be used to measure intra-abdominal depth and subcutaneous fat.

Parents’ weight and percentage body fat will be measured on a Tanita™ scale and height with a Seca™ wall-mounted stadiometer.

#### Questionnaires

A baseline questionnaire will be used to collect data from parents about demography (age, education, occupation, ethnicity, marital status), lifestyle (smoking, alcohol consumption, BMI), pregnancy (duration, weight gain), delivery and previous children (number, feeding).

We have developed and validated a questionnaire to assess milk feeding practices and hypothesised psychological mediators of any intervention effect informed by Social Cognitive Theory: attitudes, self-efficacy, outcome-expectancies and intentions with regard to following feeding recommendation (feeding and growth questionnaire) [18]. Baby eating behaviour, sleep and temperament have recently been studied as potential risk factors for obesity [19–21]. We will use a combination of questionnaires developed by other groups (Mary Rothbart and Jane Wardle) to assess these. The temperament questionnaire has been validated for use in infants [22] and the eating behaviour questionnaire has been validated in older children [23, 24].

A 4-day diet diary will be used to assess energy intake at 8 months. The diary will be the same as that used by the MRC Human Nutrition Research Unit (MRC HNR) for the National Diet and Nutrition Survey of Infants and Young Children (DNSIYC). Dietary data will be analysed using the Diet in Nutrients Out (DINO) computer package [25] by the dietary assessment team at the MRC HNR, who will not be aware of group allocation.

Parent’s quality of life will be assessed using the EuroQoL Visual Analogue Scale (VAS) and Standard Form (SF) 8. A questionnaire to measure health service utilisation (hospital, primary and community care use) using standard questions will be administered at 6 and 12 months. Parents’ perceptions of the usefulness of the different components of the intervention (face-to-face and telephone contacts, leaflets, stickers) will be assessed among intervention group participants.

The questionnaires will be self-administered prior to the visit with an opportunity to clarify any doubts during the visit.

### Randomisation and consent

Only participants who attend for the baseline visit will be randomised to intervention or control groups in order to minimise the bias from selective drop out. Central telephone randomisation will be conducted based on a random number table (generated by an independent statistician) linked to the study database. We expect that important covariates (such as birth weight, sex, gestational age, family size, maternal BMI, smoking, ethnicity, educational level, age at entry into the study and whether predominantly formula-fed) will be balanced between groups due to the large sample size. Written informed consent will be obtained at baseline visit by a trained research assistant conducting measurements.

### Intervention group

The MRC guidance for complex interventions was used to design the intervention [26]. This was an iterative process including a review of relevant theory to underpin the intervention; systematic reviews of parents’ experiences of bottle feeding [27] and determinants of non-recommended feeding practices [28]; and involvement of mothers, psychologists, doctors, midwives and health visitors [29]. The motivational component of the intervention is based on Bandura’s Social-Cognitive Theory (SCT), which has shown utility for understanding and changing dietary behaviours [30–32]. In order to help participants to translate their motivation into action, participants are encouraged to set goals and to formulate action plans, based on the concept of ‘implementation intentions’ [33–35]. They are also encouraged to monitor on a feeding-plan how much they are feeding their baby against *Baby Milk* intervention guideline amounts. Finally, participants are encouraged to keep on track by formulating coping plans (that is, problem solving), which are ‘if-then’ plans specifying how they will deal with difficult situations, such as the baby crying. In summary, the intervention consists of three components: a motivational component, an action planning component to help translate motivation into action (including goal setting, action plans and self-monitoring), and a coping planning component helping parents to deal with difficult situations.

**Fig. 1:**
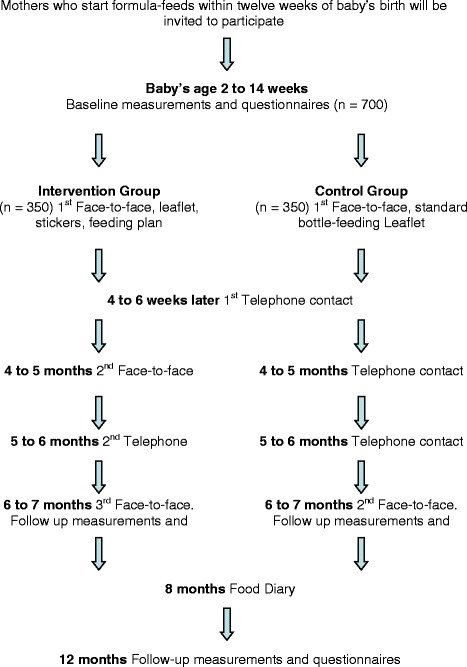
Trial Flow Chart

The intervention will be delivered by trained facilitators (research nurses) from the baby’s enrolment in the trial (2 to 14 weeks old) to 6 months and will consist of three face-to-face sessions and two telephone contacts in addition to two leaflets based on SCT (Fig. 1). The face-to-face sessions will last approximately 30 to 45 min and the telephone contacts 15 to 20 min. Since social support is important, both parents will be encouraged to attend the face-to-face sessions. During the first session, the facilitator will talk parents through the ‘healthy infant growth and nutrition’ leaflet that explains the new feeding recommendations (Table 2) and the growth charts. The leaflet does not just impart knowledge about amounts of formula-feeds, hunger cues, growth charts and rapid weight gain, but also aims to encourage recommended feeding behaviours by strengthening self-efficacy and perceived benefits about following the recommendations. Demonstration of correct feed preparation (if required) and explanation of the feeding recommendations will be provided. Parents will be encouraged to make a feeding plan and provided with stickers to put on the formula milk packets. When the baby is 4-months old, parents will be invited for a second session and advice on weaning and feedback on baby’s growth will be provided. Two phone-calls - when the baby is age 2 to 3 months and 5 months - will be made to monitor progress with following recommendations and to support the parents with ongoing goal setting, action planning, self-monitoring and problem-solving. At the 6-month visit, the baby’s growth will be plotted on the WHO growth charts. Facilitators will feed back this information to parents, and they will be encouraged to monitor the growth of their babies also after the end of the intervention delivery at 6 months. Growth monitoring is important because parents are not very good at identifying obesity in their children [36], so any programme to prevent obesity must include early identification.

**Table 2:** Comparison between the guidelines on a standard formula milk packet and *Baby Milk* intervention guidelines

		SMA guidelines		Intervention guidelines		
Age	Weight	scoops/day	kJ/kg/day	scoops/day	kJ/kg/day	% Reduction
Birth	3.5 kg	18	496	18	496	0
2 wks	3.9 kg	24	590	18	442	25
1 mo	4.4 kg	24	525	21	459	13
2 mo	5.3 kg	25	454	22	399	12
3 mo	6.1 kg	25	393	22	346	12
4 mo	6.8 kg	35	494	30	423	14
6 mo	7.8 kg	32	390	28	341	13

Based on SMA™ white: 4.4 g/scoop; 2,173 kJ per 100 g powder

### Control group

The control group will be offered the same number of contacts with facilitators (attention control) and general information about formula-feeding (bottles, teats, sterilisation, making up feeds) will be discussed. The 4 to 6 weekly phone calls will be used to discuss general issues (parenting, sleep, etcetera), to monitor feed quantity and frequency and to encourage continued participation in the study.

Parents in both groups will be given a help-line number to contact the study team about feeding issues.

### Training in intervention delivery and intervention fidelity

Facilitator training includes a 2-day training programme in the evidence base underlying the intervention, theories, behaviour change techniques, intervention strategies and communication skills, including demonstration and practice with individual feedback. The training is supported by an extensive training manual. In order to standardise intervention delivery, facilitators will use standardised protocols for all the contacts in the intervention and control conditions. Four research nurses will deliver the intervention and control group protocols and all face-to-face and telephone consultations will be audio-taped. Fidelity will be promoted, and contamination across the two groups minimised by assessing a random sample of audio-taped contacts using standardised fidelity checklists, peer appraisal, followed by feedback and team discussion. Team meetings will be held over the whole period of intervention delivery to discuss and problem solve challenging situations.

### Sample size calculation

The primary outcome is conditional infant weight gain from birth to 12 months of age (conditional on birth weight). Birth weight and weight at 6 and 12 months will be converted to standard deviation scores (SDS) adjusted for age and sex using the WHO 2006 growth standard. SDS refers to the number of standard deviations the measurement lies above (positive value) or below (negative value) the 50^th^ percentile (median value 0). Conditional weight gain is calculated as the change in weight SDS between birth to age 12 months, adjusted for birth weight SDS.

In the Avon Longitudinal Study of Parents and Children (ALSPAC), among 518 formula and mixed-fed infants, mean ± SD for conditional weight gain was 0.65 ± 0.87, and for energy intake at 4 months was 2768 ± 479 kJ/day. Based on those data, we estimated that our target 15 % (420 kJ/day) reduction in energy intake would lead to a (mean ± SE) 0.16 ± 0.04 lower gain in weight SDS between 0 to 8 months [37]. We have powered the study on a more conservative estimate of a 10 % to 11 % achieved reduction in energy intake, which corresponds to a difference in weight SDS of 0.11 to 0.12. However, the estimated effect size should be corrected for regression dilution because energy intake in ALSPAC was estimated from unweighed 1-day diet diaries. Lanigan et al. reported the within- (778 kJ) and between-infant (824 kJ) SD during the 5-day diet diaries in the 6- to 24-month-old infants, and this gives a regression dilution correction factor of 1.89 [38]. With a 10 % to 11 % lower calorie intake in the intervention group, we can expect a 0.20 to 0.21 SDS (0.11 to 0.12*1.89) difference between the two groups.

Sample sizes of 250 to 290 infants in each group will provide 80 % power at the two sided 5 % level to detect a 0.20 to 0.21 SDS difference in weight gain between the intervention and the control group. Allowing for around 15 % loss to follow-up, we aim to recruit 300 to 350 infants to each group.

### Data analysis

All quantitative data will be double-entered by an experienced, independent data entry company with whom the MRC Epidemiology Unit has an established agreement. Statistical analysis will be performed using STATA™ statistical software. All qualitative data will be transcribed verbatim and analysed using Microsoft Excel™ or the Framework™ software.

Important baseline characteristics of participants in the two groups will be described. The main analyses will compare the intervention and control groups by ‘intention-to-treat’. A secondary ‘per protocol’ analysis will be undertaken among those completing the intervention programme based on attendance at 4/5 sessions (80 % attendance). Comparisons will adjust for age, sex, birth weight, age when formula-milk was started, and other potential confounding variables. Since effects may differ between babies who are fully formula-fed and those who are mixed fed (breast and bottle), provided there are sufficient babies in the two groups, sub-group analysis for the primary outcome will be calculated within each of the two feeding practice subgroups. In addition to the primary outcome of change in weight SDS from birth to 12 months, there are a number of secondary outcomes.

#### Growth

Weight, length and BMI will be converted to SDS adjusted for age and sex using the British 1990 growth reference and the WHO 2006 growth standard at different time points (birth, baseline, six and 12 months). Babies in the intervention and control groups will be compared for change in weight, length and BMI SDS from birth and baseline to 6 and 12 months. Percentage of babies crossing the 1 percentile band (0.67 SDS) in the two groups will be compared. Their abdominal circumference, sub-cutaneous and intra-abdominal fat (skin-fold thickness and ultrasound measurements) will also be compared at 1 year.

#### Dietary intake

Differences in milk intake between intervention and control groups at 2, 3, 4, 5 and 6 months and age at introduction of solids will be compared using questionnaire data. Energy intake from 4-day diet diary data will be compared at 8 months.

#### Temperament, sleep, and eating behaviour

Between-group differences would be useful to study mediation or moderation of effect and to assess any adverse effects of our intervention on temperament and sleep.

#### Psychological mediators of infant-feeding behaviours

Differences in maternal attitudes, beliefs, intentions, self-efficacy, and outcome expectancies will be assessed between intervention and control groups. Mediation analyses will be conducted to examine to what extent any effect of the intervention on infant feeding behaviours is fully or partially explained by the above beliefs.

#### Parents’ anthropometry

A useful halo-effect of our intervention would be if due to increased awareness, parents lost weight. At 12 months, we will compare parents’ BMI between intervention and control groups, adjusted for baseline measures.

#### Acceptability

Parents’ satisfaction with different aspects of the intervention will be assessed at 6 months using a questionnaire. Semi-structured interviews in a sub-sample of intervention and control group participants and all facilitators will explore how feeding decisions were made, to explain any intervention effects, identify which components were acceptable and potentially effective, how participants responded to the intervention or control contacts, any contextual factors that influenced participant responses, and barriers and facilitators of intervention delivery as perceived.

#### Costs, health service utilisation, and economic analysis

We developed instruments to collect facilitator time spent in delivering the intervention and infant primary and secondary health service utilisation. The total cost of the intervention from the perspective of the NHS will be determined by multiplying these quantities by nationally representative unit costs. The economic evaluation will comprise a cost-consequences analysis to show the cost of delivering the intervention plus infant health service costs and outcomes (proportion of infants whose weight crosses more than one centile band upwards on the growth charts (0.67 SDS), infants of normal weight at 12 months, and probability of being normal weight as an adult using data from a meta-analysis [39]), for intervention and control groups. Where appropriate, incremental cost-effectiveness ratios will also be calculated, and uncertainty presented as 95 % confidence intervals around incremental costs and outcomes, and cost-effectiveness acceptability curves.

### Confidentiality

All information that is collected will be kept strictly confidential and stored anonymously, using a study identification (ID) number, by the MRC Epidemiology Unit, University of Cambridge and its collaborators, which includes the University of East Anglia. Codes connecting individual identity to the stored data records will be kept separately. The database containing personal information will be stored on a password and firewall protected secure standalone server, in the MRC Epidemiology Unit, University of Cambridge.

### Assessment of safety

As the aim of the intervention is to realign the growth of formula-fed infants more closely to that of breastfed infants, we consider that the risks to the babies (and parents) will be negligible. However we will actively elicit and record any (seemingly related or unrelated) adverse events and report/take action as appropriate.

#### Eliciting adverse events

Baby weight measurements might reveal underweight (weight <0.4^th^ centile) or weight faltering (crossing down through ≥2 weight centile lines). We will also ask at each visit whether the baby has had any unplanned contacts with a health professional (that is, other than routine immunisations or health checks) and ask the parent/caregiver if they have any health concerns about the baby. A potential unintended consequence of the Baby Milk intervention could be increased anxiety for the parent(s). Changes in anxiety levels will be assessed using the EuroQoL Visual Analogue Scale (VAS), completed by the primary caregiver (usually the mother) at baseline and 6-month visits. A drop in score of >30 points at 6 months will initiate a follow-up phone call by the study team.

#### Reporting adverse events

All adverse events will be recorded in the case report form, and depending on the severity and likely causality, will be reported to the Sponsor/Ethics Committee according to good clinical practice (GCP) guidelines. Incidental findings of underweight or weight faltering (as defined above) will be reviewed by our clinically trained investigators (paediatricians) and reported to the baby’s GP together with advice on management. In the first instance, in the absence of other symptoms, this will usually be more frequent monitoring of weight by the Health Visitor or GP. All other incidental findings will be discussed with clinically trained investigators who will decide whether further assessment and/or reporting to the baby’s GP, and continued participation in the trial are indicated.

## Discussion

This will be the first trial to evaluate the effectiveness and cost-effectiveness of a theory-based intervention to reduce formula-milk intake (based on the 2004 WHO recommendations for energy requirements) and prevent excess weight gain during infancy. Since 80 % of babies in England receive some formula feeds by 6 weeks of age [17], if effective, the intervention has the potential to make a significant contribution to reducing the burden of childhood obesity.

Despite the high prevalence of obesity at school entry, most prevention efforts have focussed on school-aged children and adolescents and have met with limited success [6]. Obesity prevention trials in infancy are a recent area of research because infancy is a period of rapid growth, habit formation and developmental plasticity. A 2015 systematic review of interventions delivered antenatally or within the first 2 years of life identified 27 RCTs. The 24 behavioural trials mainly focused on breastfeeding promotion, responsive feeding and parenting/maternal health while the three non-behavioural trials altered the composition of formula-milk [40]. Although these trials were designed to prevent childhood obesity, none of them specifically targeted formula-milk intake and the interventions differ markedly in their hypotheses, interventions they evaluated, design and execution from the *Baby Milk* trial.

Regardless of its efficacy in reducing energy intake and excess weight gain during infancy, the *Baby Milk* trial will enhance understanding of participant and contextual factors that influence excess infant feeding, an area that is currently under-researched. The results of this study will also be informative to the Scientific Advisory Committee on Nutrition (SACN) and Department of Health with regard to the future adoption of the revised WHO guidelines in the UK.

## Trial status

The trial was recruiting at the time of submission.

## Abbreviations

ALSPAC: Avon Longitudinal Study of Parents and Children
BMI: body mass index
DH: Department of Health
DINO: Diet In Nutrients Out
DNSIYC: Diet and Nutrition Survey of Infants and Young Children
GCP: good clinical practice
GP: general practice
NCMP: National Childhood Measurement Programme
NHS: National Health Service
MRC: Medical Research Council
MRC HNR: Medical Research Council Human Nutrition Research Unit
SACN: Scientific Advisory Committee on Nutrition
SCT: Social Cognitive Theory
SDS: standard deviation score (number of standard deviations above or below the 50^th^ percentile)
VAS: visual analogue scale, measure of health status on a scale of 1–100
WHO: World Health Organisation
